# Phosphodiesterase 7 inhibitor reduces stress-induced behavioral and cytoarchitectural changes in C57BL/6J mice by activating the BDNF/TrkB pathway

**DOI:** 10.3389/fphar.2024.1411652

**Published:** 2024-07-18

**Authors:** Jiahao Dong, Ran Wei, Fangjiao Zong, Zhe Wang, Shengyao Ma, Wei Zhao, Yuanyuan Lin, Aixin Zhang, Ge Lan, Fang Zhang, Han-Ting Zhang

**Affiliations:** Department of Pharmacology, Qingdao University School of Pharmacy, Qingdao, China

**Keywords:** phosphodiesterase 7, stress exposure, single prolonged stress, synaptic plasticity, cAMP signaling

## Abstract

**Background:**

Phosphodiesterase 7 (PDE7) plays a role in neurological function. Increased expression and activity of PDE7 has been detected in several central nervous system diseases. However, the role of PDE7 in regulating stress levels remains unclear. Thus, this study aimed to determine whether and how PDE7 involved in the stress-induced behavioral and neuron morphological changes.

**Methods:**

The single prolonged stress (SPS) was used to build a stress exposure model in C57BL/6 J mice and detected PDE7 activity in hippocampus, amygdala, prefrontal cortex and striatum. Next, three doses (0.2, 1, and 5 mg/kg) of the PDE7 inhibitor BRL-50481 were intraperitoneally administered for 10 days, then behavioral, biochemical, and morphological tests were conducted.

**Results:**

PDE7 activity in hippocampus of mice significantly increased at all times after SPS. BRL-50481 significantly attenuated SPS induced anxiety-like behavior and fear response in both context and cue. In addition, BRL-50481 increased the levels of key molecules in the cAMP signaling pathway which were impaired by SPS. Immunofluorescent staining and Sholl analysis demonstrated that BRL-50481 also restored the nucleus/cytoplasm ratio of hippocampal neurons and improved neuronal plasticity. These effects of BRL-50481 were partially blocked by the TrkB inhibitor ANA-12.

**Conclusion:**

PDE7 inhibitors attenuate stress-induced behavioral changes by protecting the neuron cytoarchitecture and the neuronal plasticity in hippocampus, which is mediated at least partly through the activation of BDNF/TrkB signaling pathway. These results proved that PDE7 is a potential target for treating stress-induced behavioral and physiological abnormalities.

## 1 Introduction

Post-traumatic stress disorder (PTSD) is a complex chronic mental disorder characterized by distress related to trauma-related cues and impaired fear extinction. It is usually triggered by life-threatening events, such as natural disasters, wars, sex harassment, domestic violence, and serious traffic accidents (American Psychiatric Association, 2013; Henigsberg et al., 2019; Yabuki and Fukunaga, 2019). At present, the mechanisms underlying the pathogenesis of PTSD remain unclear while the development of potential therapeutic strategies remains an urgent clinical requirement (Krediet et al., 2020). Stress susceptibility is one determinant of mental disorders, individuals with high stress sensitivity are often more susceptible to developing PTSD. Additionally, patients tend to maintain a high level of stress during the disease (Jeong et al., 2020; Skórzewska et al., 2020). Therefore, it is of great significance to study the physiological changes after stress and identify effective interventions for regulating stress levels. The hippocampus plays a pivotal role in the formation and consolidation of memory, as well as in regulating emotions, fear, anxiety, and stress (Giotakos, 2020). Previous research has indicated that severe stress often results in damage to the neuronal structure and function in the hippocampus, as well as interference with the expression of key signaling molecules (Kim et al., 2015; McEwen et al., 2016). Thus, restoring neuronal damage induced by stress may be an effective strategy to reduce stress levels.

Cyclic adenosine monophosphate (cAMP) is one of the most important secondary messengers in cells. It participates in numerous physiological and pathological processes of the body (Azevedo et al., 2014; Delhaye and Bardoni, 2021). Activating the downstream protein kinase A (PKA) signaling pathway is one of the major ways in which second messengers exert their effects, thereby activating and phosphorylating cAMP-response element binding protein (CREB) (Fertig and Baillie, 2018). CREB phosphorylation plays a regulatory role in neuronal growth, survival, and synaptic connectivity, further impacting the function of the hippocampus (Lonze and Ginty, 2002). Specifically, CREB can control the synthesis of specific proteins, including brain-derived neurotrophic factor (BDNF), an important biological molecule for maintaining the normal function of neurons (Pak et al., 2021; Lu et al., 2022). Further, previous studies found that BDNF plays an important regulatory role in neuron growth and differentiation by specifically binding to its receptor, tyrosine kinase receptor B (TrkB) in the hippocampus, meanwhile, the hormone level changes resulting from severe stress exposure have a regulatory effect on the BDNF/TrkB signaling pathway (Notaras and van den Buuse, 2020; Camuso et al., 2022). In stressed model mice, BDNF expression is decreased in the hippocampus, accompanied by severe fear conditioning responses and inefficient extinction of fear memories (Peters et al., 2010; Ni et al., 2020; Zhang et al., 2021).

Phosphodiesterases (PDEs) are a superfamily of enzymes containing 11 isoenzymes encoded by 21 different genes. PDEs regulate a variety of intracellular signal transduction pathways, particularly through cAMP and cGMP (Delhaye and Bardoni, 2021). PDEs play an important role in regulating the functions of the central nervous system (CNS), as well as PDE inhibitors have anti-inflammatory, antioxidant, vasodilator, antidepressant, and memory-enhancing effects (Sadeghi et al., 2023). In addition, many studies have expanded the correlation between PDE4 and the pathogenesis of CNS diseases, demonstrating the beneficial effects of PDE4 inhibitors on CNS diseases such as stroke, Alzheimer’s disease, and depression (Richter et al., 2013; Blokland et al., 2019). Nevertheless, the side effects induced by PDE4 inhibitors (notably nausea and vomiting) greatly limit their clinical utility (Schick and Schlegel, 2022). PDE7, another cAMP-specific PDE, is widely distributed in the CNS. However, while PDE7 inhibitors have similar neuroprotective effects to those of PDE4 inhibitors (Zorn and Baillie, 2023), unlike PDE4 inhibitors, PDE7 inhibitors do not cause serious adverse reactions such as nausea and vomiting (García et al., 2014). Thus, PDE7 inhibitors are promising candidates for promoting neuron survival, improving cognitive function, and treating memory deficits (Chen et al., 2021). However, the effects of PDE7 inhibitors on stress level and the underlying mechanisms have not been investigated so far.

To investigate whether PDE7 inhibitors have potential regulatory effects on stress levels, we adopted a controlled, prospectively modified single prolonged stress (SPS) model (Lisieski et al., 2018; Richter-Levin et al., 2019). The regime included restraint, forced swimming, ether anesthesia, and foot shock. This way, mice can be induced to exhibit anxiety-like behavior and severe fear conditioning responses with high modeling rates (Feng et al., 2020; Ni et al., 2020; Xi et al., 2021). We determined the changes of PDE7 activity in different brain regions, and the effects of PDE7 inhibitor on stress-induced behavioral and cytoarchitectural changes of mice. We also investigated the effect of PDE7 inhibitor on key signaling molecules in cAMP pathway to determine the specific regulatory mechanism. The present results provide valuable insights into the regulation of stress level and support the potential of PDE7 inhibitors as a therapeutic approach for stress exposure-related diseases.

## 2 Materials and methods

### 2.1 Animal preparation and modified SPS model

Adult male C57BL/6 mice (8–12 weeks old, 25.0 ± 2.0 g) were maintained on a 12:12 h light–dark cycle under an ambient temperature of 22–25°C with food and water *ad libitum*. Single prolonged stress and electrical stimulation was conducted as described previously (Wang et al., 2008). Briefly, mice were initially immobilized for 2 h in a restraint tube, immediately followed by forced swimming for 20 min in an acrylic cylindrical tank (50 cm × 30 cm) filled with water (20–24°C). A 15-min recovery period, mice were exposed to ether vapor until loss of consciousness (<1 min), after a 30-min rest, mice were subjected to a single shock (2 mA, 2 s) in an electric shock box before being returned to home cages.

### 2.2 Experimental procedures and drug administration

BRL-50481 (Selleck, Lot. S5837) was dissolved in 0.9% sterile saline containing 2.5% dimethyl sulfoxide (DMSO), and administered once per day for 10 days via intraperitoneal injection (i.p.) at doses of 0.2, 1, and 5 mg/kg. ANA-12 (Selleck, Lot. S774501) was dissolved in 0.9% sterile saline containing 2.5% DMSO at 0.5 mg/kg/day. Behavioral tests were performed 30 min after the last drug treatment. The control and model groups received the same amount of saline (containing equivalent amounts of DMSO). After modeling or treatments, mice were anesthetized with 5% isoflurane, decapitated, and hippocampal tissues were dissected on ice.

### 2.3 PDE7 activity determination by HPLC

The activity of PDE7 was measured according to the method published by Monzel et al. with slight modifications (Montoya et al., 2014; Monzel et al., 2014). Ater mice were euthanized, the hippocampus, amygdala, prefrontal cortex and striatum were quickly separated and weighed. Each tissue was homogenized with 1 mg of tissue 20 μL Radio-Immunoprecipitation Assay (RIPA) Lysis Buffer, and the supernatant was collected after centrifugation. The consumption of cAMP was determined using an external standard method. Each sample was divided into three equal parts for the following treatment, Part a: 20 μL of inactivated enzyme samples, 60 μL of cAMP (250 μM), and 20 μL of PBS were mixed to a final volume of 100 μL; Part b: 20 μL of sample, 60 μL of cAMP (250 μM), and 20 μL of PBS were mixed to a final volume of 100 μL; Part c: 20 μL of sample, 60 μL of cAMP (250 μM), and 20 μL of BRL-50481 (500 μM) were mixed to a final volume of 100 μL. Then the three parts mixture were incubated at room temperature for 50 min, followed by heating at 100°C for 5 min to denature the proteins in the sample and terminate the hydrolysis reaction of PDE. The mixtures were centrifuged to collect the supernatant and impurities are filtered out with a 0.22 μm filter. Then the Cyclic adenosine monophosphate (cAMP) was determined by high performance liquid chromatography (HPLC) as described in the Supplementary Material, and the PDE7 activity was calculated by the consumption of cAMP in each sample.

### 2.4 Behavioral tests

#### 2.4.1 Open field test

The open field test (OFT) was used to measure locomotion and evaluate the anxiety level. Performing the experiment in a dimly lit room enforces the natural exploration behavior of mice. Mice were placed at the center of a cubic chamber (400 mm (W) × 400 mm (D) × 500 mm (H)) and allowed to explore freely for 10 min. A camera positioned atop the device recorded the movements and the average speed of mice, which were used to analyze the time and the distance explored in central area as a parameter of its anxiety level.

#### 2.4.2 Elevated plus maze test

The elevated plus maze (EPM) test was also used to evaluate anxiety-like behavior of SPS mice. The apparatus consisted of two opposite-facing open arms (350 (W) mm × 50 (D) mm × 150 (H) mm), two opposite-facing closed arms (350 (W) × 50 (D) × 150 (H) mm), and a central area (50 (W) × 50 (D) mm), which was elevated 500 mm above the floor. The device was surrounded by a single background curtain. At the beginning of each round of testing, the mouse was placed in the central area facing the closed arm, and allowed to explore freely on the device for 5 min. A camera located directly above the device recorded movements of mice, which were used to and analyze the number of times mice entered open arm and the time mice explored in open arm as a parameter of its anxiety level.

#### 2.4.3 Fear conditioning response (FCR) test

Contextual and cued fear conditioning paradigms, consisting of a training and two test sessions, were used to detect fear conditioning response. Training sessions were executed on day 13 after SPS. Mice were placed in the contextual chamber for 180 s to familiarize themselves with the environment, then presented with a tone (28 s, 1 kHz, 90 dB) immediately followed by a foot shock (2 s, 0.8 mA). The tone-foot shock cycle was repeated three times. After the cycle ended, mice were allowed to rest for 2 min in the chamber. Then, the mice were returned to their home cages. After 24 h, mice were placed in the contextual chamber for 5 min without exposure to tone or foot shock to test contextual fear response. The cued fear response test was conducted 1 h later. The mice were placed in a novel chamber with a changed appearance for 3 min to adapt to the environment. Then, a tone (4 kHz, 80 dB) was presented for 3 min without foot shocks. Mice continued to rest in the chamber for 1 min before returning to the home cages. Mice activities were captured by a built-in camera in the device, and the freezing time was calculated and analyzed using the VISU TRACK software.

### 2.5 Immunofluorescent staining

On day 14 post-SPS, mice were anesthetized and perfused with 0.9% physiological saline through the heart, followed by fixation with 4% paraformaldehyde. Brain tissue was cut into paraffin-embedded blocks and sectioned into 5 µm slices using a paraffin microtome. The slices were incubated in an oven at 60°C for 2 h, dewaxed, and dehydrated. Antigen retrieval was performed using 1 × Tris EDTA (pH = 9.0). Then, slices were rinsed twice with PBS and treated with 3% endogenous peroxidase, BSA + 0.3% TritonX-100 at room temperature for 15 min. Brain sections were then incubated with the primary antibody anti-NeuN (1:800, Sevicebio) overnight at 4°C. After washing with PBS three times, the slices were incubated with the secondary antibodies Alexa fluor 568 Goat anti rabbit IgG for 1 h at room temperature in the dark. Next, slices were incubated with DAPI for 30 min and sealed with an anti-fluorescence quenching agent. Images were acquired using a confocal microscope. ImageJ software was used for analysis.

### 2.6 Golgi staining and sholl analysis

Brains were stained using the FD Rapid Golgi Stain commercial kit (FD Neurotechnologies, Ellicot City, MD, United States). Brains were placed in the impregnation solution (composed of a mixture of equivalent volumes of Solution A and Solution B) prepared 24 h in advance, and incubated at room temperature in the dark for 14 days. Subsequently, the brain tissue was transferred to Solution C and incubated for 3 days. Based on the stereotactic map of the mouse brain, coronal sections (150 µm) of the hippocampus were obtained using a cryostat. The collected slices were treated with a mixed solution of Solution D and Solution E for 10 min at room temperature. Then, washed with double distilled water and dehydrated with ethanol of different concentrations. The sections were then washed in xylene three times and covered using coverslips. Next, the dendritic branches originating from ten intact CA1 pyramidal neurons per animal were traced and reconstructed. All traced neurons belonged to the same anatomical position in the CA1 subregion of the hippocampus. To analyze the morphogenesis of the dendritic branch, Sholl analysis was performed using the Neuron J plugin in ImageJ software, as previously reported (Qiu et al., 2014). Briefly, we plotted a series of concentric circles centered on the soma (10 µm radius increment from the soma) overlapping with the pyramidal neurons to quantify the dendritic tracings, including the total dendritic length, branching complexity, and branch point locations, and to detect regional alterations in the dendritic arbor structure.

### 2.7 Western blotting

Western blotting was performed according to a standard protocol (Molecular Clone, Edition II). Twenty micrograms of total protein from tissues were resolved using SDS-PAGE and transferred to PVDF membranes. The following primary antibodies were used: anti-BDNF (1:1,000, Abcam); anti-PSD95 (1:5,000, Abcam); anti-Synaptophysin (1:10,000, Abcam); anti-p-TrkB (1:1,000, Abcam); anti-TrkB (1:2000, Abcam); anti-p-CREB (Ser133, 1:1,000, Cell Signaling); and anti-CREB (1:1,000, Cell Signaling). All blots were detected using an enhanced chemiluminescence detection system (Advansta). The scanned images were quantified using ImageJ.

### 2.8 ELISA

cAMP levels in the hippocampus were determined using ELISA. Hippocampal tissues were obtained, weighed, tissue was homogenized with 1 mg of tissue and 20 μL of PBS, and fragmented using a high-throughput tissue grinder. After centrifugation at 3,000 rpm for 15 min at 4°C, the supernatant was collected, and the protein concentration determined using the BCA Kit. A cAMP ELISA kit (ML057902, ML BIO) was used to determine the cAMP levels of hippocampus according to manufacturer’s instructions. Absorbance was detected at 450 nm before calculating the cAMP concentration based on a standard curve.

### 2.9 Statistical analysis

All data are presented as mean ± standard error of the mean (SEM). Statistical analyses were performed using GraphPad Prism 8.0 software (GraphPad Inc., United States). Data were statistically analyzed by one-way or two-way ANOVA followed by Bonferroni test. The *p*-values <0.05 were considered statistically significant differences.

## 3 Results

### 3.1 PDE7A’s enzymatic activity significantly increased in stress exposed mice

**FIGURE 1:**
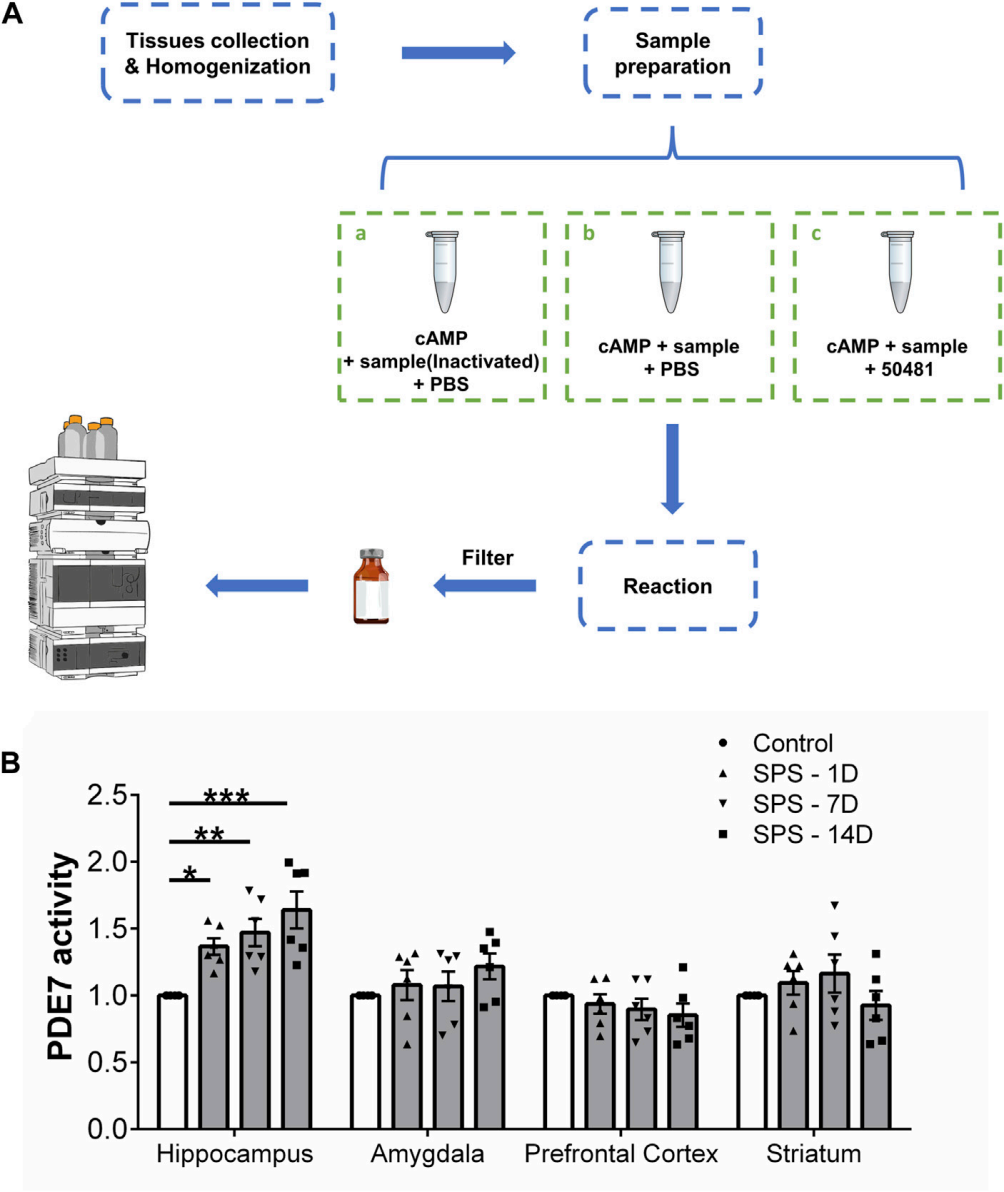
PDE7 activity in different brain regions of mice at 1, 7, and 14 days after SPS induction. **(A)** Experimental scheme for measuring cAMP by HPLC. **(B)** Relative activity of PDE7 in hippocampus, amygdala, prefrontal cortex and striatum of mice. Data are presented as mean ± SEM (n = 6 mice per group). ^*^ *P* < 0.05, ^**^ *P* < 0.01, ^***^ *P* < 0.001 vs. control.

We evaluated the changes of PDE7 activity in the hippocampus, amygdala, prefrontal cortex and striatum of mice at 1, 7, and 14 days after SPS induction (Figure 1A). Figure 1B shows that SPS induced a significant increase in PDE7 activity in the hippocampus (F (3, 20) = 8.728, *p* = 0.0007), which increased gradually with modeling time. However, no significant changes in PDE7 activity were found in other brain regions (amygdala, F (3, 20) = 0.9764, *p* = 0.4235; prefrontal cortex, F (3, 20) = 0.8142, *p* = 0.5011; striatum, F (3, 20) = 1.092, *p* = 0.3752) after SPS induction. Therefore, SPS induced behavioral or physiological changes may be related to the increased PDE7 activity in hippocampus.

### 3.2 PDE7 inhibitor reduced severe fear conditioning responses and anxiety-like behaviors in stress exposed mice

**FIGURE 2:**
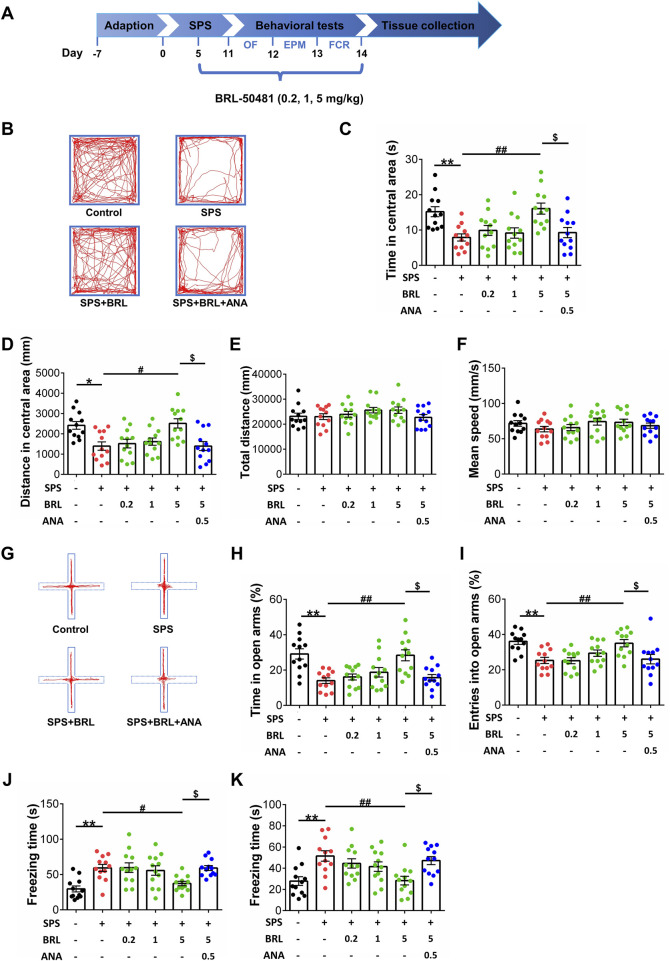
PDE7 inhibitor ameliorated the anxiety-like behaviors in OFT and EPMT as well as the severe fear conditioning response in FCRT after SPS induction. **(A)** Experimental schedule for the modified single prolonged stress model and behavioral tests. Representative trajectories in OFT **(B)** and EPMT **(G)** of each group. Time **(C)** and distance **(D)** in the central area of OFT. Mean speed **(E)** and total distance **(F)** in OFT. Duration time **(H)** and entry times **(I)** on the open arms in EPMT. Freezing time in contextual **(J)** and cued **(K)** fear conditioning response. Data were presented as mean ± SEM (n = 12 mice per group). ^*^ *P* < 0.05, ^**^ *P* < 0.01 vs. control; ^#^ *P* < 0.05, ^##^ *P* < 0.01 vs. SPS; ^$^ *P* < 0.05 vs. BRL 5-treated SPS.

To verify the effect of PDE7 inhibition on severe fear conditioning response and anxiety-like behavior induced by SPS in mice, we performed the behavioral tests indicated in Figure 2A. Namely, OF, EPM, and FCR tests. In the OFT, the movement trajectory of mice in the open field chamber is shown in Figure 2B, as shown in Figure 2C (F (5, 66) = 6.175, *p* < 0.0001) and D (F (5, 66) = 6.226, *p* < 0.0001), SPS induced a significant decrease in the Figure 2C time and distance explored of mice in central area, and treatment with BRL-50481 at 5 mg/kg significantly increased both the time and distance explored in central area thereby reducing the anxiety-like behaviors induced by SPS. Figure 2E (F (5, 66) = 1.180, *p* = 0.3282) and F (F (5, 66) = 0.9956, *p* = 0.4274), show that there were no differences in total distances and average speed between each group of mice in the OFT, suggesting that the locomotor capacity of mice was not influenced by SPS. In the EPM test, the movement trajectory of mice in the elevated plus-maze is shown in Figure 2G, as shown in Figure 2H (F (5, 66) = 7.867, *p* < 0.0001) and I (F (5, 66) = 6.251, *p* < 0.0001), SPS induced a significant decrease in the entries time and the explored time in open arms of mice. At 5 mg/kg, BRL-50481 significantly increased the entries time and time explored in the open arms. Similar to what we observed in the OFT, the anxiety-like behavior of SPS mice was ameliorated by PDE7 inhibition. In the FCR, as shown in Figure 2J (F (5, 66) = 6.799, *p* < 0.0001) and K (F (5, 66) = 5.290, *p* = 0.0004), SPS exposure significantly increased both contextual and cue fear responses, i.e., increased the freezing time. At 5 mg/kg, BRL-50481 significantly decreased the freezing time. Similar to the OF and EPM tests, BRL-50481 at 5 mg/kg reduced severe fear conditioning responses. To further test whether BRL-50481 reduced the behavioral changes by activating the BDNF/TrkB pathway, we used ANA-12 to block TrkB activity. The improvement effects of BRL-50481 were blocked by ANA-12, indicating that the PDE7 inhibitor reduced fear conditioning responses and anxiety-like behaviors in SPS mice at least partially through the BDNF/TrkB pathway.

### 3.3 PDE7 inhibitor activates BDNF/TrkB signaling by increasing cAMP levels in the hippocampus of stress exposed micee

**FIGURE 3:**
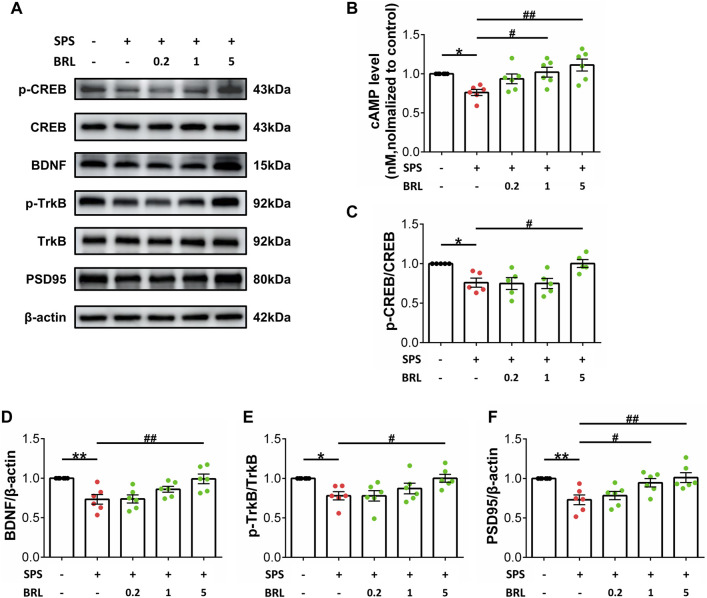
PDE7 inhibitor ameliorated SPS induced BDNF and PSD95 downregulation as well as CREB and TrkB inactivation in hippocampus by increasing cAMP levels. **(A)** Representative blots of the molecular expression in hippocampal tissue of mice **(B)** cAMP level in the hippocampal tissue. Relative quantitative ratio of p-CREB/CREB **(C)** p-TrkB/TrkB **(E)** in the hippocampal tissue. Quantifications of BDNF **(D)** PSD95 **(F)** in the hippocampal tissue. Data are presented as mean ± SEM (n = 5 or 6 mice per group). ^*^ *P* < 0.05, ^**^ *P* < 0.01 vs. control; ^#^ *P* < 0.05, ^##^ *P* < 0.01 vs. SPS.

Our experiments showed increased PDE7 activity in the hippocampus after SPS, and PDE7 inhibitors could improve the behavioral changes induced by SPS. Next, we determined the effect of PDE7 inhibitor on cAMP levels and the downstream key signaling molecules p-CREB/CREB, BDNF, p-TrkB/TrkB, and PSD95 expression (Figure 3A). Figure 3B (F (4, 25) = 5.625, *p* = 0.0023) shows that the cAMP level in the SPS group was significantly lower than that in the control group, and that treatment with BRL-50481 (1, 5 mg/kg) could increase cAMP levels in the hippocampus of SPSmice. As shown in Figure 3C (F (4, 20) = 6.026, *p* = 0.0024) and D (F (4, 25) = 7.369, *p* = 0.0005), SPS exposure significantly decreased the ratio of p-CREB to CREB and BDNF expression. In addition, SPS exposure significantly decreased the p-TrkB to TrkB ratio (F (4, 25) = 4.423, *p* = 0.0077) and PSD95 expression (F (4, 25) = 6.329, *p* = 0.0012) (Figure 3E, F). Conversely, treatment with 5 mg/kg BRL-50481 significantly promoted BDNF and PSD95 expression in the hippocampus of SPS mice as well as increased p-CREB and p-TrkB levels. Interestingly, 1 mg/kg BRL-50481 was sufficient to increase PSD95 expression.

### 3.4 PDE7 inhibitor ameliorated the impairment of neuronal cytoarchitecture in hippocampal CA1 subregion induced by SPS

**FIGURE 4:**
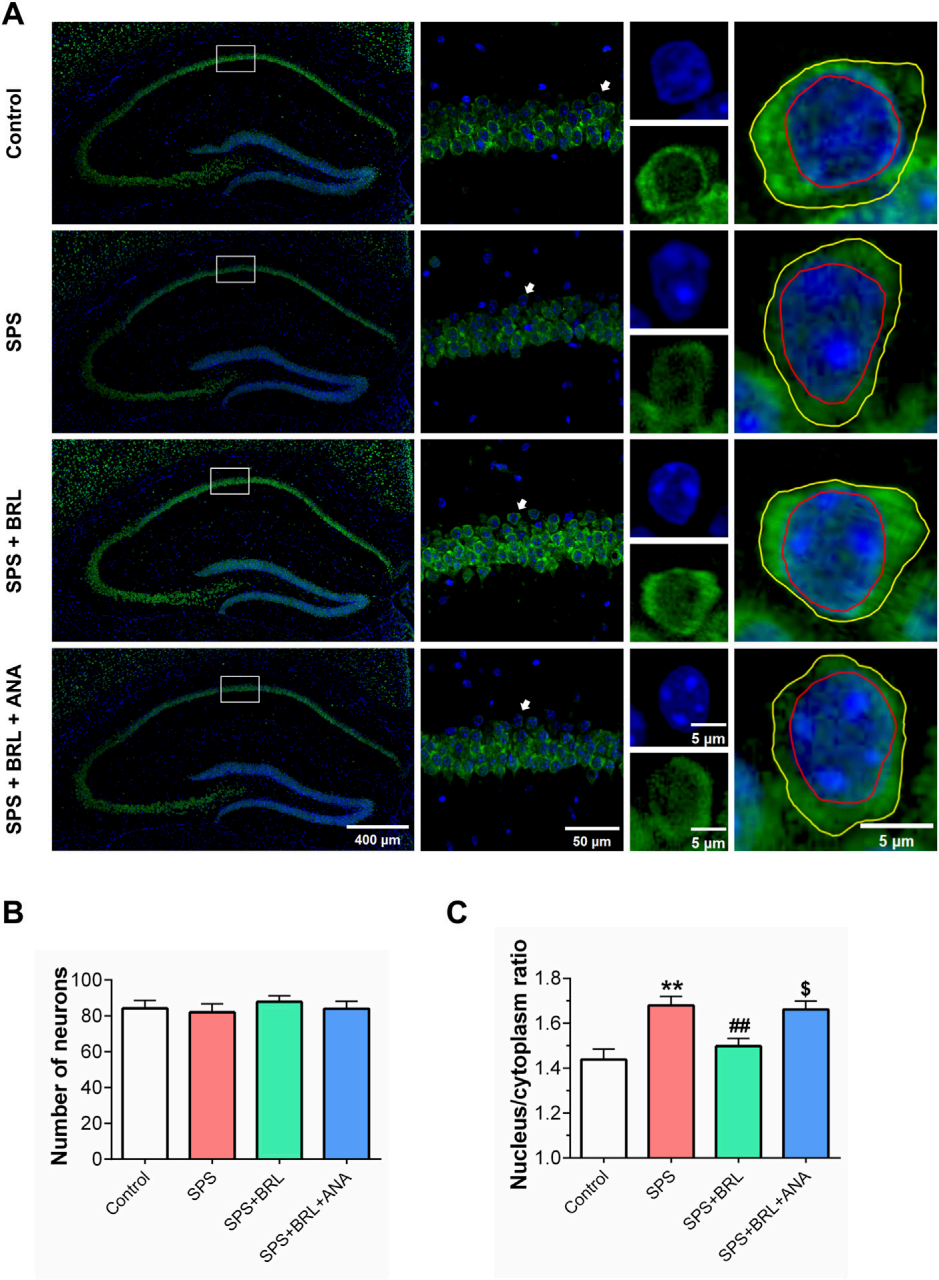
PDE7 inhibitor ameliorated the increased nucleus/cytoplasm ratio of neurons in hippocampal CA1 induced by SPS. **(A)** Representative images of the nucleus and cytoplasm of the hippocampal neurons after illustrated with anti-NeuN (green) and DAPI (blue) immunofluorescent staining. Number of hippocampal CA1 neurons **(B)**, and nucleus/cytoplasm ratio of hippocampal CA1 neurons **(C)**. Data were presented as mean ± SEM (n = 24 neurons/4 mice per group). ^**^ *P* < 0.01 vs. control; ^##^ *P* < 0.01 vs. SPS; ^$^ *P* < 0.05 vs. SPS + BRL.

**FIGURE 5:**
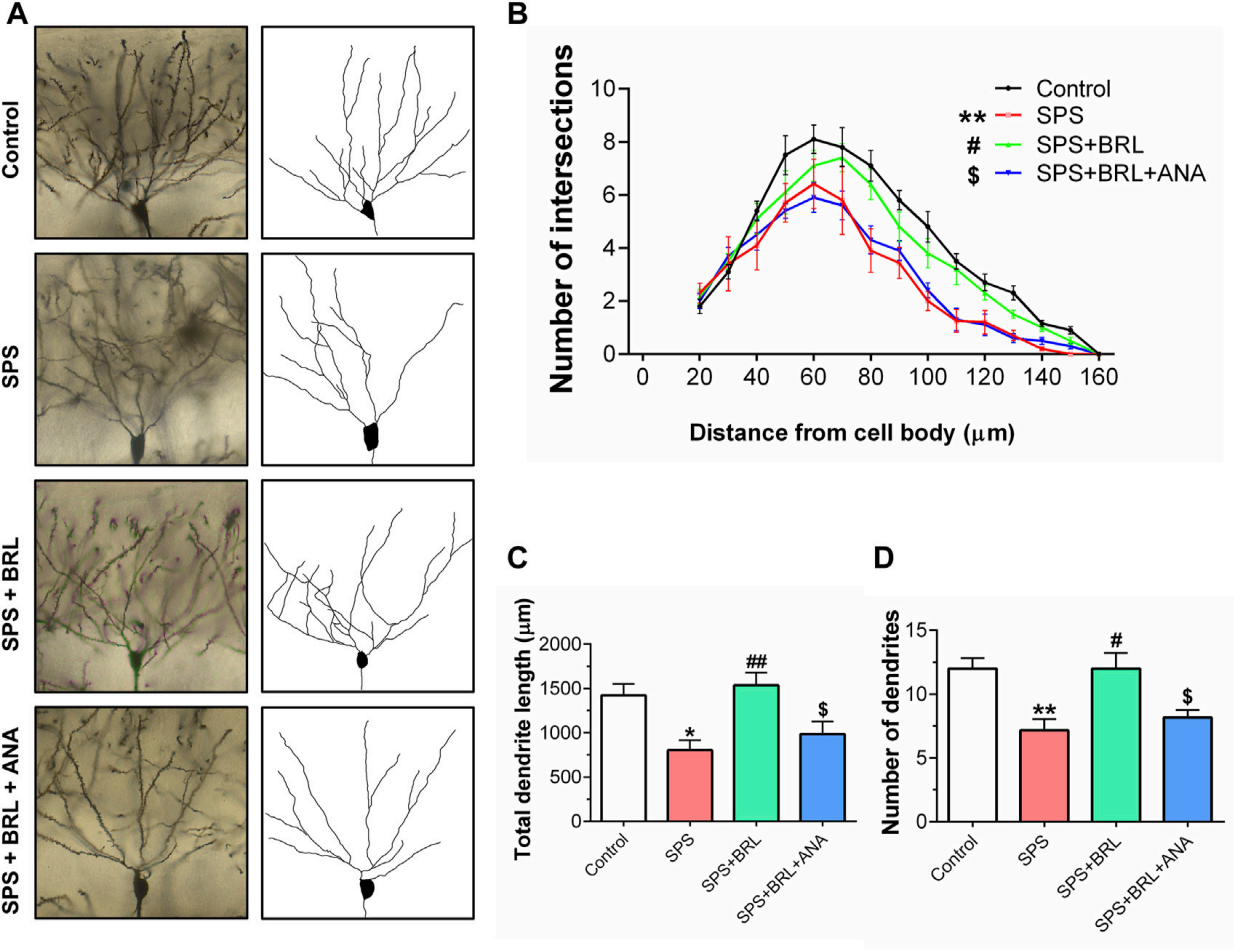
PDE7 inhibitor ameliorated the impairment of dendritic structure in hippocampal neurons. **(A)**. Representative images of neuronal dendrites labeled with Golgi impregnation. **(B)**. Sholl analysis of the hippocampal neurons revealed the alterations of basal dendritic intersections at distinct distances from soma. The total dendritic length **(C)**, and dendritic numbers **(D)** of neurons. Data were presented as mean ± SEM (n = 36 neurons/6 mice per group). ^*^ *P* < 0.05, ^**^ *P* < 0.01 vs. control; ^#^ *P* < 0.05, ^##^ *P* < 0.01 vs. SPS; ^$^ *P* < 0.05 vs. SPS + BRL.

On day 14 after SPS induction, we detected the morphological changes of neurons in hippocampal CA1 by immunofluorescence staining (Figure 4A). Using anti-NeuN to label the soma of neurons, NeuN (Hexaribonucleotide Binding Protein-3), as a biomarker of neurons, can specifically label the nucleus and cytoplasm of neurons. Using DAPI to label the nucleus to distinguish the nucleus and cytoplasm by different colors. Then, the soma and nucleus of neurons were outlined by ImageJ software to analyze the nucleus/cytoplasmic ratio. No significant changes were observed in the numbers of hippocampal CA1 neurons (F (3, 92) = 1.555, *p* = 0.2058) after SPS (Figure 4B). However, SPS exposure significantly increased the ratio of nucleus/cytoplasm in neurons (Figures 4C; Figures 5C, F (3, 92) = 8.932, *p* = 0.0006). The normal neurons typically have a stable nuclear/cytoplasmic ratio, when neurons suffer damage, the cytoplasm collapses, causing an increase in the nucleus/cytoplasm ratio. Treatment with 5 mg/kg BRL-50481 could normalized the nucleus/cytoplasm ratio in neurons. In addition, ANA-12 significantly increased the nucleus/cytoplasm ratio. The increase in the ratio of nucleus/cytoplasm indicated the degeneration of neurons, which might further lead to neuronal dysfunction. These results indicated that BRL-50481 has a recovery effect on the impaired cytoarchitecture of CA1 neurons; since ANA-12 blocked this recovery effect, PDE7 inhibitors probably protected CA1 neurons by activating the BDNF/TrkB pathway, similar to the improved effects on behavioral abnormalities.

### 3.5 PDE7 inhibitor ameliorated SPS induced deficits in dendritic arborization complexity

To further study the effect of the PDE7 inhibitor on the plasticity of hippocampal neurons, the dendritic arborization of CA1 neurons was assessed using Golgi staining at day 14 post-SPS. Representative images of CA1 pyramidal neurons and their reconstructed dendritic arborization are shown in Figure 5A. The number of points where the basal dendritic intersects with the virtual Sholl shell in a Sholl analysis, was counted as a parameter the complexity of dendrites. As shown in Figure 5B (F (3, 140) = 9.800, *p* = 0.0003), SPS induced a significant decrease in dendritic intersections, which was markedly improved with BRL-50481 (5 mg/kg) treatment. As expected, ANA-12 significantly blocked the improvement effect. In addition, SPS induced a significant decrease in the total length (Figure 5C, F (3, 140) = 6.906, *p* = 0.0034) and the number (Figure 5D, F (3, 140) = 7.737, *p* = 0.0013) of dendrite. All these negative changes induced by SPS were reduced by BRL-50481 treatment while can be blocked by ANA-12. These results indicate that the treatment with BRL-50481 enhanced the dendritic complexity of hippocampal neurons after SPS by activating the BDNF/TrkB signaling, thereby contributing to reduce the stress level of mice after exposure.

## 4 Discussion

**FIGURE 6:**
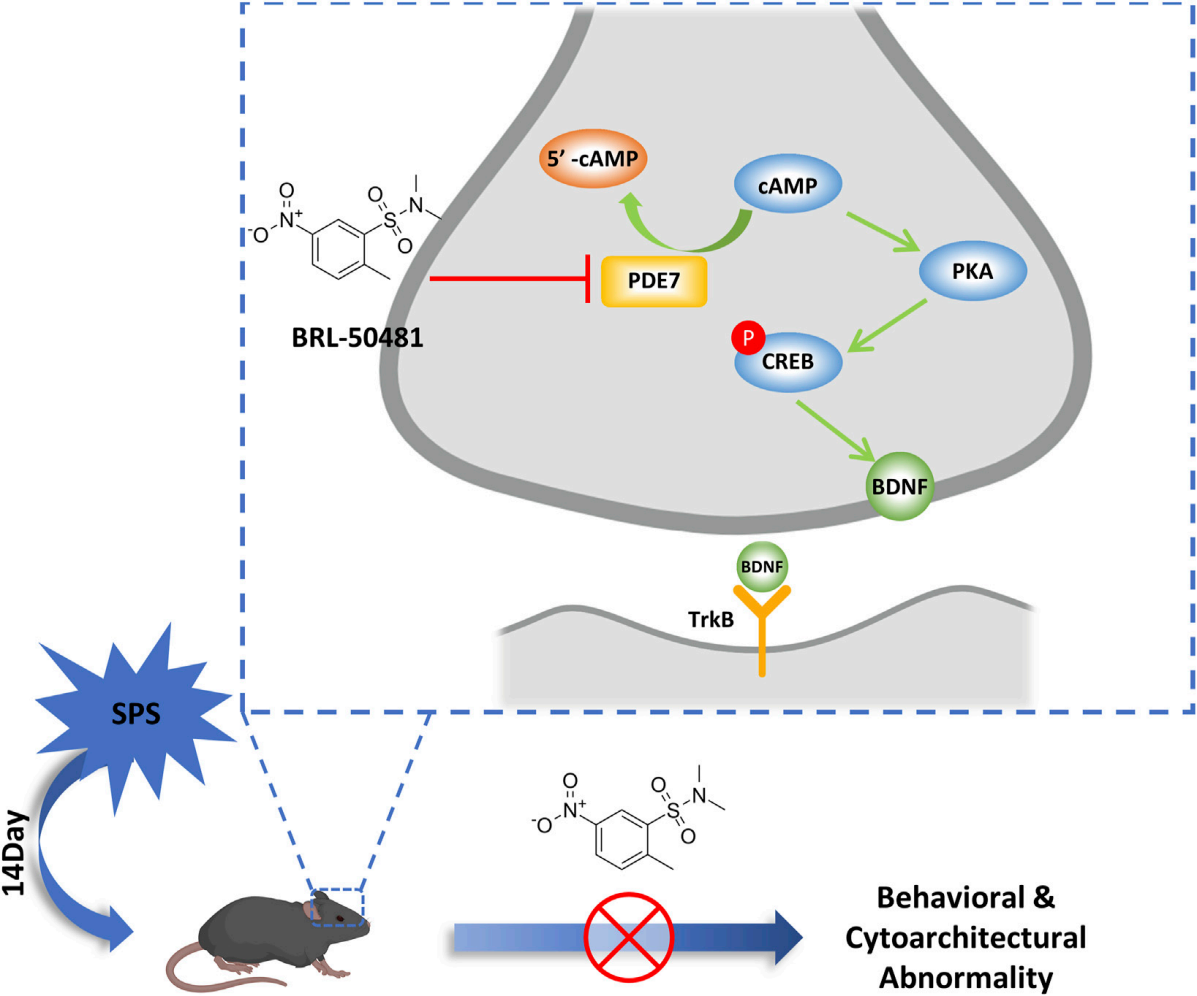
Schematic representation of the mechanism of PDE7 inhibitor improving the behavioral and cytoarchitectural abnormality in SPS mice. PDE7 inhibition reduces severe fear conditioning responses and anxiety-like behavior in SPS mice, meanwhile repairing the neuron damage in hippocampus, and these improvement effects were achieved by the activation of BDNF/TrkB pathway.

This study provides preclinical evidence that PDE7 inhibitors can alleviate stress induced behavioral changes by repairing the damage of neuronal cytoarchitecture and synaptic plasticity. These improved effects were associated with upregulation of cAMP and its downstream BDNF/TrkB signaling (Figure 6).

SPS is a classic model for studying stress-related diseases, due to its modeling process contains multiple stress stimulations, resulting in more stable and durable changes in both behavioral and physiological of mice (Yamamoto et al., 2010; Knox et al., 2016; Feng et al., 2020). Therefore, the SPS was employed in the present study to provide acute and severe stressors for mice. The mice exhibited anxiety-like behavior and severe fear conditioning response in 14 days after SPS. The factors inducing these phenotypes may be the excessive pressure caused by restraint, forced swimming, electric shock and other means in SPS process, damaging the normal morphological and functional properties of neurons as well as critical molecular signaling pathways (Perrine et al., 2016; Lisieski et al., 2018). We conducted PDE7 enzyme activity assays in brain regions which strongly implicated in the regulation of stress level. As a specific cAMP hydrolase, the changes of PDE7 activity may produce the influence on the structure and function of hippocampus and further affect the behavioral characterization of animals by affecting the level of cAMP (Leal et al., 2014). Our study first identified that PDE7 activity increased in the hippocampus of mice after SPS, whereas that in other brain regions, including the amygdala, striatum, and prefrontal cortex, remained unchanged. These findings suggest that the behavioral changes induced by stress exposure may be related to the increase of PDE7 activity in hippocampus. Interestingly, although no changes in PDE7 activity were found in the amygdala after SPS, the results showed that PDE7 inhibitor had an effect on the non-hippocampus-dependent cued fear response. Undeniably, stress exposure affects not only the hippocampus and amygdala, but also the entire brain of mice, while the impact on the hippocampus is relatively severe, it cannot be ruled out that other brain regions may also play a role in behavioral dysfunction of mice. There may be multiple interpretations of the results. One hypothesis is that PDE7 inhibitors act on the hippocampus and subsequently influence the amygdala through interactions with other brain regions, thereby showing effects on cued fear response. Another hypothesis is that while PDE7 activity in the amygdala is not affected by stress exposure, actively reducing PDE7 activity in the amygdala can still play a regulatory role in amygdala-dependent cued fear conditioning response.

Exposure to repeated and severe emotional stressors can lead to structural and functional damage in the hippocampus (McEwen and Magarinos, 1997; Giotakos, 2020). Sustained high level of stress caused by trauma event could impair the structure and biochemical molecules of neurons, ultimately leading to the behavioral abnormalities (Shucard et al., 2012; Farrell et al., 2015; Guo et al., 2022). Therefore, targeting the signaling molecular pathways involved in hippocampal function protection is an effective strategy for regulating stress levels. The regulatory effect of PDE7 on the nervous system is mainly through its regulation of cAMP level. In the CNS, cAMP activates CREB phosphorylation and controls BDNF synthesis by affecting gene transcription (Bollen and Prickaerts, 2012; Giralt et al., 2013; Nthenge-Ngumbau and Mohanakumar, 2018). In this study, we found that the expression of BDNF in hippocampus of mice was decreased after SPS, PDE7 inhibitor could ameliorate the decrease of cAMP levels, CREB phosphorylation, and BDNF expression. After blocking BDNF/TrkB pathway with ANA-12, the improvement effect of PDE7 inhibitor was also blocked. BDNF is an important neurotrophic factor in the brain, and its functions include influencing the growth and development of neurons and the plasticity of synapses (Zagrebelsky and Korte, 2014; Camuso et al., 2022). Previous studies have shown that a genetic or environmental deficiency in BDNF-TrkB signaling could lead to improper development and maintenance of hippocampal volume and plasticity, potentially resulting in heightened stress susceptibility in individuals (Green et al., 2013; Notaras and van den Buuse, 2020). As anticipated, the PDE7 inhibitor improved neuronal damage in the hippocampus by increasing BDNF expression and further activating downstream TrkB receptors, thereby reducing the stress-induced behavioral changes of mice. Therefore, the activation of the BDNF/TrkB pathway may represent a critical mechanism by which PDE7 inhibitors exert improvement effects on hippocampal neurons.

The normal morphology of neurons is the basis for maintaining their normal physiological function. Prolonged stress exposure can result in morphological damage to hippocampal neurons in mice, leading to a neuronal dysfunction (Dow et al., 2015; Besnard and Sahay, 2016). The present study supports these findings and further revealed that the structural damage of neurons in the hippocampal CA1. Although the study results showed no decrease in the total number of neurons, the increase of the nucleus/cytoplasm ratio in CA1 neurons suggests that the soma of neurons have collapsed, which may be related to neuronal dysfunction. Previous studies have shown that the morphology of dendrites in neurons has a profound impact on the function of synaptic, the bigger a dendritic tree, the more synapses it can receive and the more presynaptic cells and synapses it can sample, which will enhance the ability to receive and integrate synaptic information (Sjöström et al., 2008; Lefebvre et al., 2015). However, abnormal morphological damage in dendrites could reduce the computational ability of neurons, leading to neurobehavioral abnormalities (Wong, 2008). In our results, stress exposure resulted in damage to the dendritic structure of hippocampal neurons in mice and reduced the expression of PSD-95. Since the PSD-95 is one of the most abundant postsynaptic proteins and its enhanced expression can drastically increase synaptic strength (Ehrlich and Malinow, 2004; Zhang and Lisman, 2012). BRL-50481 improved the expression of PSD-95 and repaired the damage of dendritic arborization complexity in neurons, increasing the total length and total number of dendrites, while these improvement effects could be partially blocked by ANA-12, indicating that enhancing synaptic structure and PSD-95 expression, thereby protecting synaptic plasticity in hippocampus, is related to the activation of BDNF/TrkB signaling pathway. Collectively, we provided solid demonstrations that PDE7 is a potential target for regulating stress-induced behavioral and cytoarchitectural changes, and these regulatory effects at least partly related to the BDNF/TrkB pathway.

## 5 Conclusion

In the present study, we provided reliable demonstrations that BRL-50481 ameliorated stress induced anxiety-like behaviors and severe conditioned fear responses in SPS mice by increasing the level of cAMP and activating the BDNF/TrkB pathway in hippocampus. Meanwhile, BRL-50481 reduced the damage of neuronal cytoarchitecture in hippocampal CA1 region and improved dendritic complexity deficits in SPS mice. Our findings support the development of selective PDE7 inhibitors to target the repair of neuronal damage for the treatment of stress exposure-related diseases.

## 6 Limitations

1) In this study, we lacked administrating of PDE7 inhibitors to the control mice, so the effects of PDE7 inhibitors on SPS mice in the results may be reversing the pathological changes of SPS, or it may be changing baseline in the opposite direction of the SPS effect. (2) It cannot be completely ruled out that BRL-50481 has off-target effects, so the improvement caused by BRL-50481 is partly but not entirely due to the increase in cAMP levels resulting from its inhibition of PDE7.

## Data Availability

The original contributions presented in the study are included in the article/Supplementary Material, further inquiries can be directed to the corresponding authors.
