# Supplementary material for: Phosphodiesterase 7 inhibitor reduces stress-induced behavioral and cytoarchitectural changes in C57BL/6J mice by activating the BDNF/TrkB pathway
Source: Front Pharmacol. 2024 Jul 18;15:1411652. doi: 10.3389/fphar.2024.1411652 (PMC11291325; doi:10.3389/fphar.2024.1411652)
Supplement: Supplementary file 1 [file DataSheet2.PDF]

Phosphodiesterase 7 inhibitor reduces stress-induced behavioral and cytoarchitectural changes in C57BL/6J mice by activating the BDNF/TrkB pathway

Supplementary Methods:

1. Quantitation of cAMP by HPLC

According to the method published by Monzel et al. with slight modifications. The Cyclic adenosine monophosphate (cAMP) was determined by high performance liquid chromatography system (1200 series, Agilent, Waldbronn, Germany), consisting of a binary pump (G7111A), an autosampler (G71116A), and a DAD-detector (G71115A). For determination of cAMP, an Agilent 5 TC-C18 (Agilent Technologies, Böblingen, Germany) chromatographic column (250 × 4.6 mm) was used. The eluent consisted of acetonitrile and potassium dihydrogen phosphate in a ratio of 6:94. with a flow rate of 1.2 mL/min and a column temperature of 25℃.Sample injection volume was 20 μl. Wavelength of the cAMP detection is 254 nm. Retention time was at the sixth minute. After measuring the peak area, the consumption of cAMP in a. b and c is calculated based on the standard curve.

Supplementary Tables:

Suppl. Table 1: The average peak areas (mA·min) of cAMP in the a. b and c parts of the samples of each group measured by HPLC (n=6).

	Control			SPS-1D			SPS-7D			SPS-14D		
Hippocampus	a	b	c	a	b	c	a	b	c	a	b	c
	515	191	**252**	521	197	**278**	517	199	**287**	511	190	**289**
Amygdala	a	b	c	a	b	c	a	b	c	a	b	c
	522	272	315	527	277	319	514	261	302	520	255	299
Cortex	a	b	c	a	b	c	a	b	c	a	b	c
	513	171	221	533	163	209	523	169	211	519	167	203
Striatum	a	b	c	a	b	c	a	b	c	a	b	c
	528	332	372	513	315	357	525	309	355	526	330	366

Supplementary Figures:

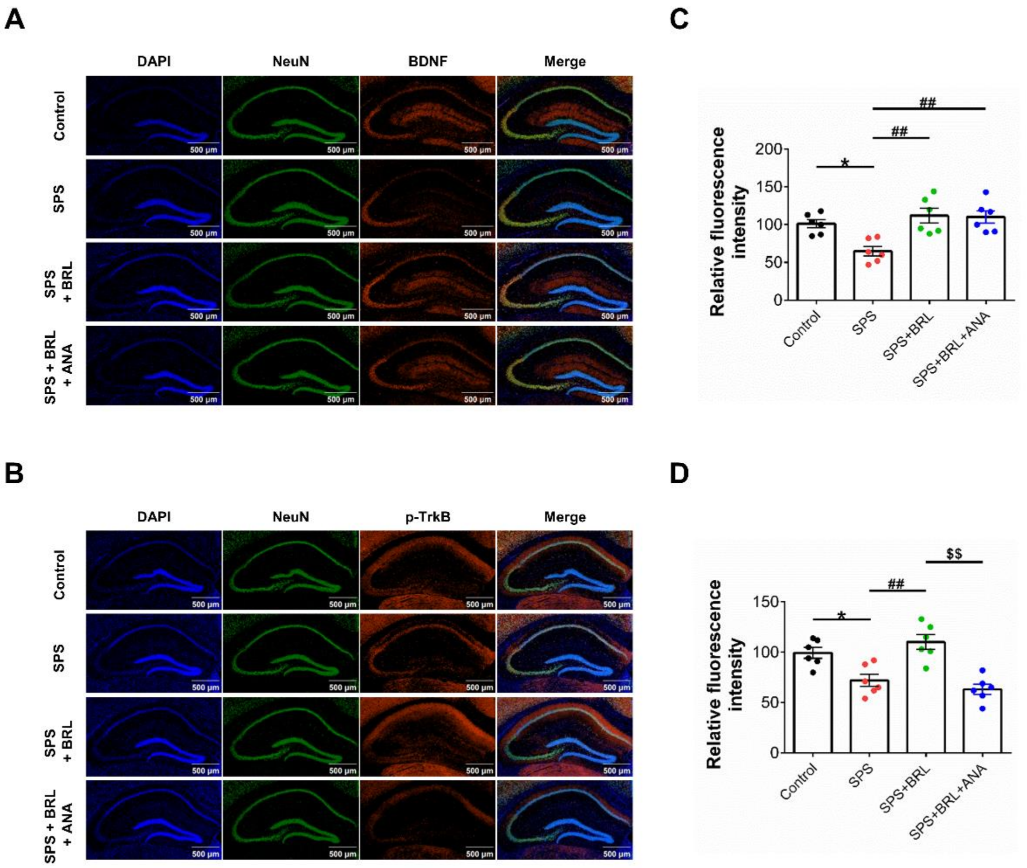

Suppl. Fig. 1: PDE7 inhibitor reduced BDNF and p-TrkB downregulation induced by SPS in hippocampal tissue. A-B. Tissue were stained with anti-BDNF (red) or anti-p-TrkB (red) antibodies, nucleus were stained with DAPI (blue), nurons were stained with Neun (green) and the staining results were observed by confocal. Relative fluorescence intensity of BDNF (C) and p-TrkB (D) in hippocampus. Data were presented as mean ± SEM (n = 4 mice per group). $^{*}P < 0.05$ *vs*. control; $^{\#\#}P < 0.01$ *vs*. SPS; $^{\$\$}P < 0.01$ *vs*. BRL 5-treated SPS.

References:

Göttel, C., Niesen, S., Daub, V., Werle, T., Bakuradze, T., Winterhalter, P., et al. (2020). In Vitro Inhibition of Phosphodiesterase 3B (PDE 3B) by Anthocyanin-Rich Fruit Juice Extracts and Selected Anthocyanins. *Int J Mol Sci* 21, 6934. doi: 10.3390/ijms21186934

Montoya, G. A., Bakuradze, T., Eirich, M., Erk, T., Baum, M., Habermeyer, M., et al. (2014). Modulation of 3’,5’-cyclic AMP homeostasis in human platelets by coffee and individual coffee constituents. *Br J Nutr* 112, 1427–1437. doi: 10.1017/S0007114514002232

Monzel, M., Kuhn, M., Bähre, H., Seifert, R., and Schneider, E. H. (2014). PDE7A1 hydrolyzes cCMP. *FEBS Lett* 588, 3469–3474. doi: 10.1016/j.febslet.2014.08.005

Pöch, G. (1971). Assay of phosphodiesterase with radioactively labeled cyclic 3',5'-AMP as substrate. *Naunyn Schmiedebergs Arch Pharmakol* 268, 272–299. doi: 10.1007/BF00997262

Röhrig, T., Pacjuk, O., Hernández-Huguet, S., Körner, J., Scherer, K., and Richling, E. (2017). Inhibition of Cyclic Adenosine Monophosphate-Specific Phosphodiesterase by Various Food Plant-Derived Phytotherapeutic Agents. *Medicines (Basel)* 4, 80. doi: 10.3390/medicines4040080

Spoto, G., Fioroni, M., Rubini, C., Di Nicola, M., Di Pietrantonio, F., Di Matteo, E., et al. (2004). Cyclic AMP phosphodiesterase activity in human gingival carcinoma. *J Oral Pathol Med* 33, 269–273. doi: 10.1111/j.0904-2512.2004.00092.x
