# Supplementary material for: Phosphodiesterase 7 inhibitor reduces stress-induced behavioral and cytoarchitectural changes in C57BL/6J mice by activating the BDNF/TrkB pathway
Source: Front Pharmacol. 2024 Jul 18;15:1411652. doi: 10.3389/fphar.2024.1411652 (PMC11291325; doi:10.3389/fphar.2024.1411652)

Supplementary Figure 1. Original western blot for p-CREB and CREB (43kDA)

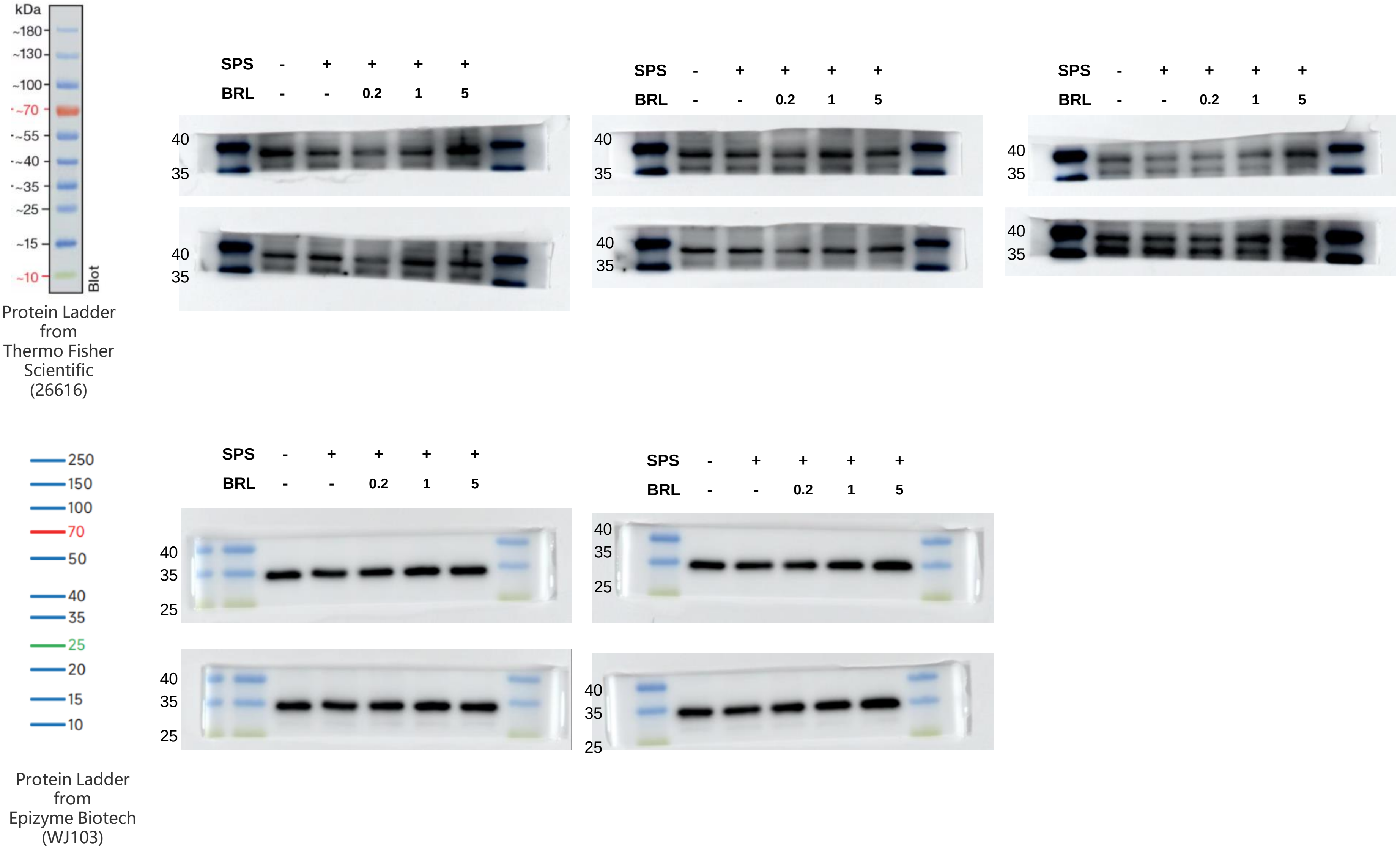

Supplementary Figure 2. Original western blot for BDNF (15kDA) and β-actin (42kDA)

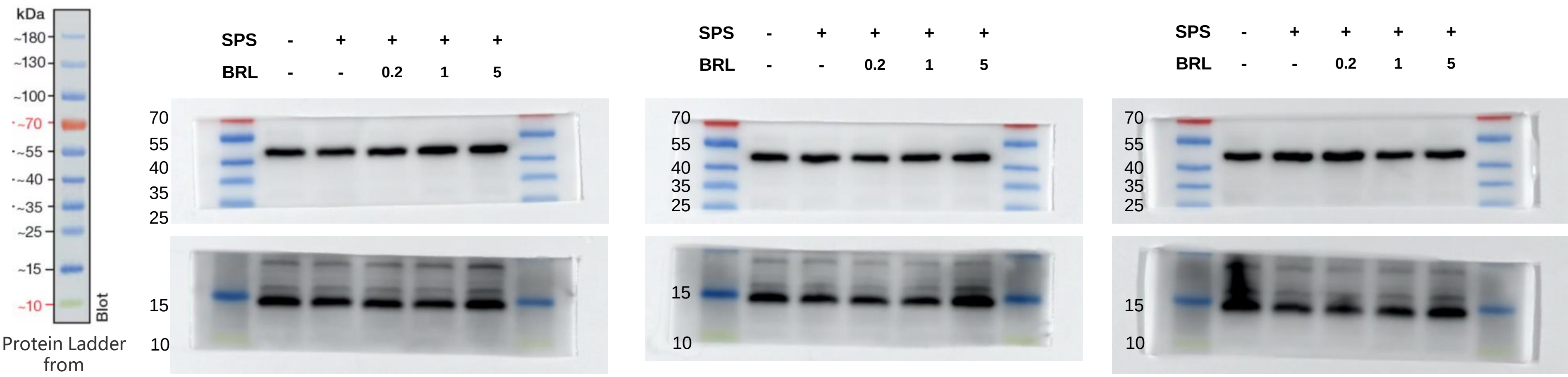

Protein Ladder from Thermo Fisher Scientific (26616)

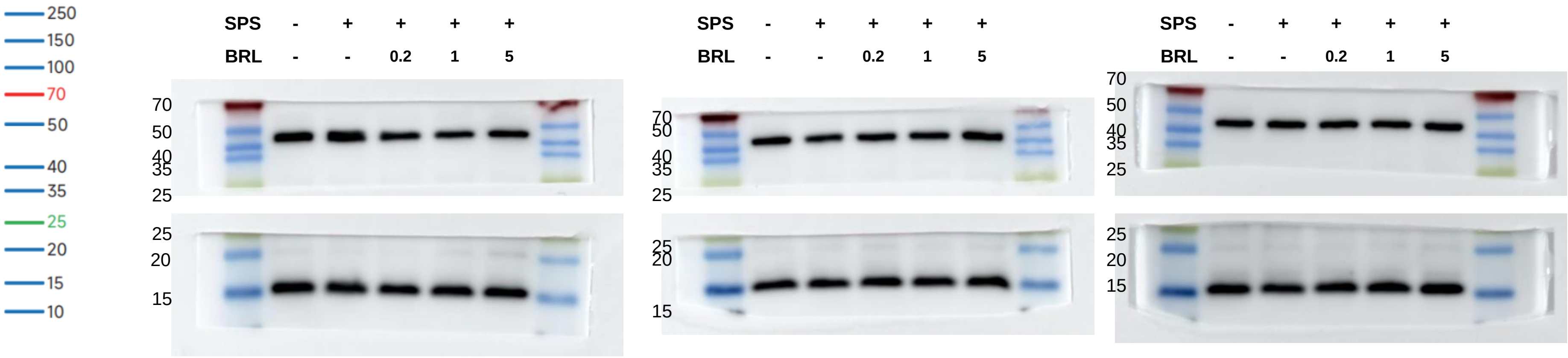

Protein Ladder from Epizyme Biotech (WJ103)

Supplementary Figure 3. Original western blot for p-TrkB and TrkB (92kDA)

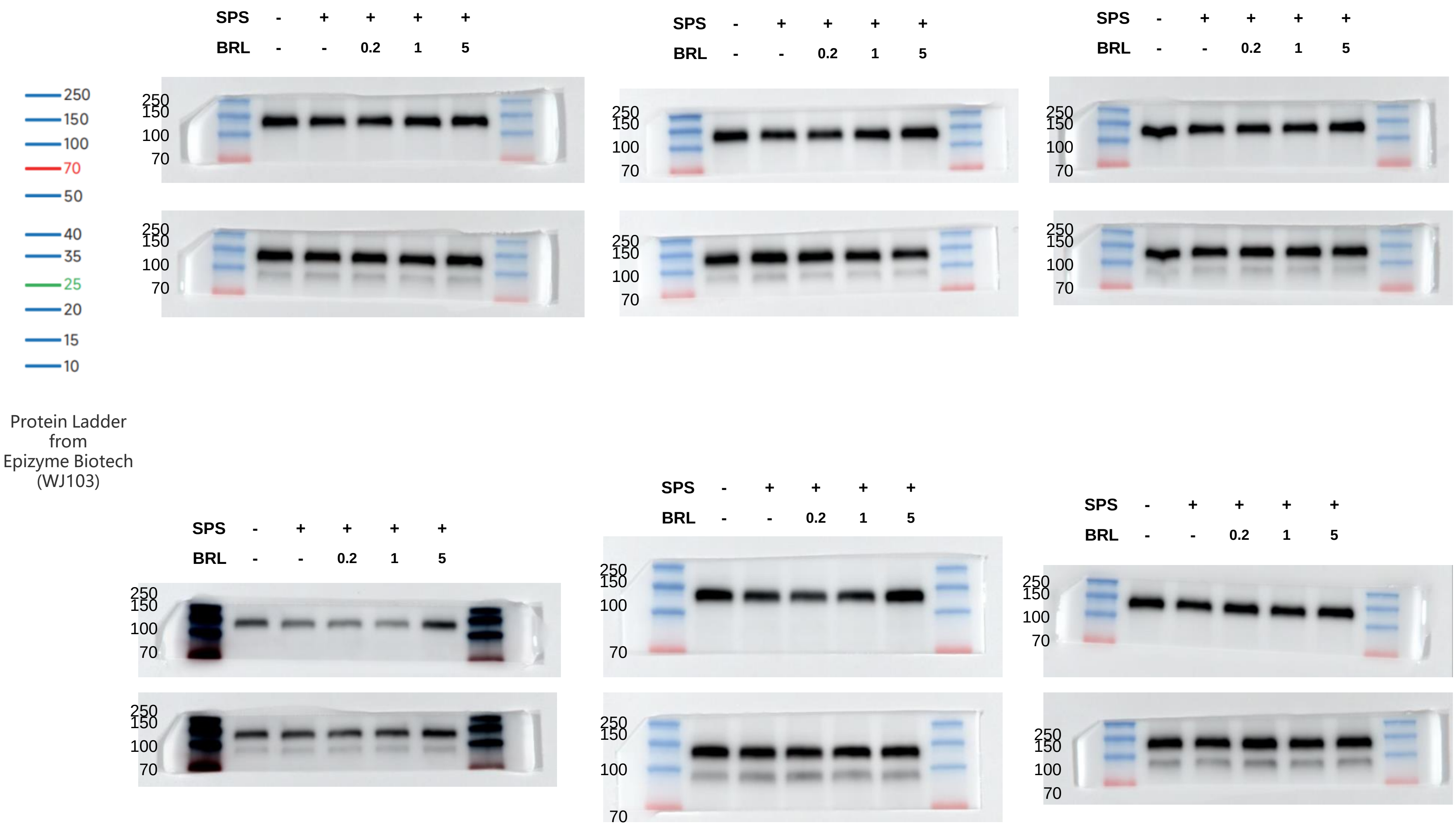

Supplementary Figure 4. Original western blot for PSD95(80kDA) and Tubulin(55kDA)

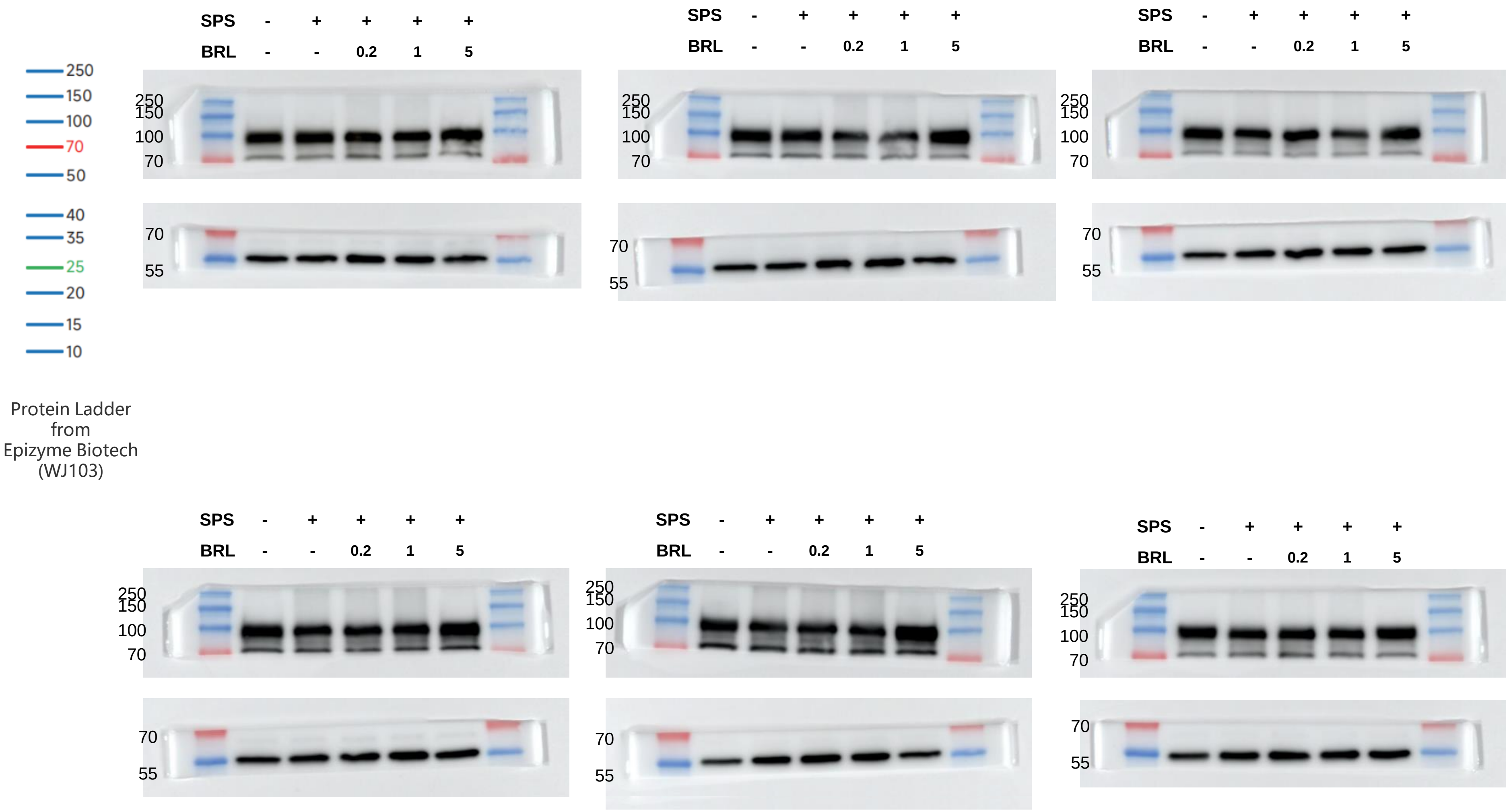

Supplement: Supplementary file 2 [file DataSheet1.PDF]
